# Is Cardiac Resynchronisation Therapy Proarrhythmic?

**Published:** 2008-11-01

**Authors:** Francisco Leyva, Paul WX Foley

**Affiliations:** Department of Cardiology, University of Birmingham, Good Hope Hospital, West Midlands, England.

**Keywords:** cardiac resynchronisation therapy, arrhythmias, mortality, heart failure

## Abstract

It is well established that cardiac resynchronisation therapy (CRT) using biventricular pacing  prolongs survival by its effects on pump failure. The rate of sudden cardiac death in patients undergoing CRT, however, remains high. Animal and human studies have shown that reversal of normal sequence of myocardial activation during epicardial pacing, as applied during CRT, increases the transmural dispersion of repolarisation (TDR), a substrate for ventricular arrhythmias. Cohort studies in humans suggest that CRT has a differential effect on the arrhythmogenic substrate, antiarrhythmic in some and proarrhythmic in others. This review the focuses on the possibility that CRT may, under certain circumstances, promote arrhythmogenesis.

Cardiac resynchronisation therapy (CRT) using biventricular pacing (BiVP) has revolutionised the treatment of heart failure. In the Cardiac Resynchronization Heart Failure (CARE-HF) study, which included patients with with a QRS complex ≥120 ms, NYHA class III or IV and a left ventricular ejection fraction of ≤35%, CRT was associated with a 36% reduction in all-cause mortality [1]. This and other studies have shown that CRT also leads to an improvement in NYHA class, walking distance, quality of life and reverse left ventricular remodelling [2-5].

The rate of sudden cardiac death remains high in patients with heart failure who are treated with CRT [6]. In the first report of the CARE-HF study, CRT did not reduce the rate of sudden cardiac death [1]. In the Comparison of Medical Therapy, Pacing, and Defibrillation in Heart Failure (COMPANION) study, CRT with defibrillator back-up (CRT-D) led to a significant reduction in mortality [4], suggesting that ventricular arrhythmias as the cause of death in some patients with heart failure undergoing CRT.

Increasing evidence indicates that the myocardium is electrically and mechanically heterogenous. Reversal of normal sequence of myocardial activation following epicardial pacing, as it occurs during CRT, has been shown to increase the TDR, a substrate for ventricular arrhythmias [7,8]. These issues have raised concern as to whether CRT is proarrhythmic. This review explores the effects of epicardial pacing on the arrhythmogenic substrate and their relevance to the possible proarrhythmic effects of CRT.

## Electrical heterogeneity of the myocardium

**Figure 1:**
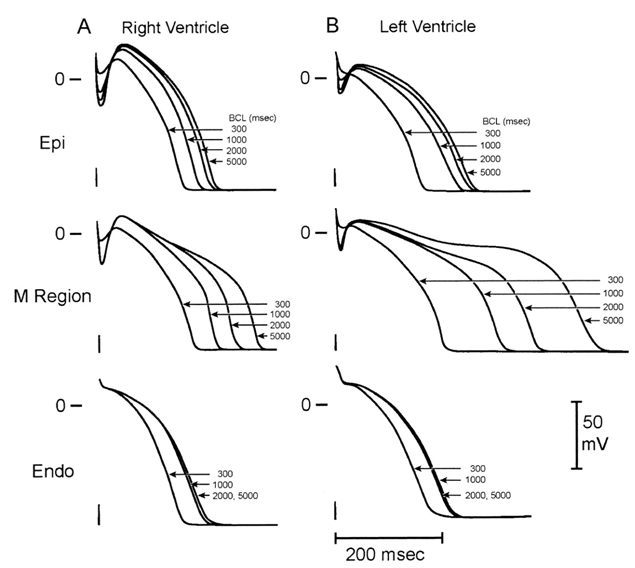
M-cell action potential duration in response to slowing of the stimulation rate. Transmembrane recordings were obtained from epicardial (Epi), endocardial (Endo) portions of the canine right and left ventricles at cycle lengths of 300, 1,000, 2,000 and 5,000 ms. Modified and reprinted with permission from Lippincott Williams & Wilkins [9].

The concept of electrical heterogeneity in the myocardium arose from a seminal study in 1991, in which Sicouri and Antzelevitch described a subpopulation myocardial cells with distinct electrophysiological properties, known as M cells [9]. Amongst its characteristics, the M cell generates an action potential which prolongs to a greater degree than that of the epicardium and endocardium when the heart rate slows [9,10]. (Figure 1) Electrical heterogeneity has been demonstrated in various animal species and in the human myocardium [11].

## Electrical heterogeneity and the surface ECG

Surrogate measures of electrical heterogeneity can be found in the12-lead ECG, which is, in effect, a single image of electrical activity of the myocardium propagated in three dimensions. The QT interval is a macroscopic measure of ventricular repolarisation which reflects, ultimately, the cellular action potential. The unipolar chest leads from V1 to V6 more accurately reflect local activity [12] and, for this reason, these leads are used preferentially in the assessment of QT dispersion.

The morphology of the T wave reflects voltage gradients generated by epicardial, endocardial and M layers. The peak of the T wave corresponds to full repolarisation of the epicardial action potential, whereas the end of the T wave coincides with repolarisation of the M cells. The length of the QT peak interval therefore depends on the duration of the epicardial action potential, whereas the QT interval duration reflects the duration of the M-cell action potential.

The Tpeak-Tend interval provides a measure of the transmural dispersion of repolarisation (TDR) [8,13-16]. A prolonged T_peak_-T_end_ has been linked to spontaneous development of ventricular tachycardia and, interestingly, with increased inducibility [17]. In effect, a prolonged T_peak_-T_end_ marks the presence of an arrhythmogenic substrate.

Although QT dispersion has been shown to predict mortality in population based studies [18], and in patients with myocardial infarction [19] and left ventricular dysfunction [20-22], it has generally been disappointing as a predictor of arrhythmic events.

## Electrical effects of epicardial pacing

**Figure 2:**
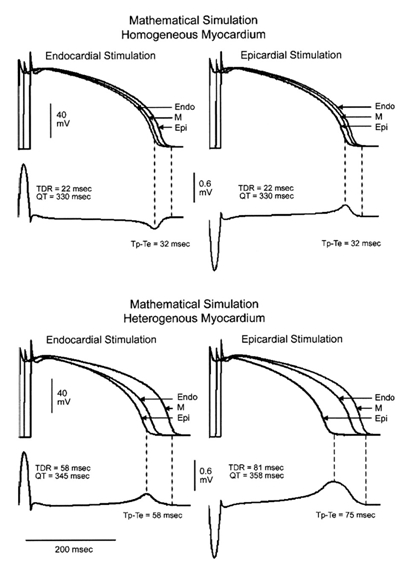
Top: Effects of epicardial (Epi) versus endocardial (Endo) pacing on QT interval, T_peak-end_ (T_p_-T_e_) and TDR in homogenous myocardium and in heterogenous myocardium. Note that, when switching from endocardial to epicardial pacing, the QT interval, T_peak-end_ (T_p_-T_e_) and TDR are unchanged in homogenous myocardium but prolonged in heterogenous myocardium. Modified and reprinted with permission from Lippincott Williams & Wilkins [8].

The QT interval, the morphology of the T wave and the T_peak_-T_end_ interval are dependent on the activation sequence of the myocardium. In an elegant study using mathematical modelling, Fish et al [8] demonstrated the difference between transmural conduction in homogenous versus heterogenous myocardium. (Figure 2) In homogenous myocardium, reversing of the direction of stimulation leads to a change in the polarity of the QRS complex and the T wave, but not to changes in the duration of the action potential, the QT interval, TDR or the T_peak_-T_end_ interval. In heterogenous myocardium consisting of epicardium, endocardium and M cells, reversal of the transmural direction of activation does lead to a prolongation of TDR and the T_peak_-T_end_ interval, reflecting the fact that the epicardium depolarises and repolarises earlier and the M calls depolarise and repolarise later.

Bai et al studied the effects of epicardial LV pacing and biventricular pacing in an experimental model of dilated cardiomyopathy in dogs [23]. In control dogs, the mean action potential duration was prolonged in the three layers of the myocardium, being shortest in the subepicaridum and longest in the mid layer. Left ventricular epicardial and biventricular pacing in controls dogs was associated with a prolonged transmural dispersion of repolarisation, compared to RV endocardial pacing. The surface ECG showed that LV epicardial pacing resulted in a longer T_peak_-T_end_ compared to RV endocardial pacing. In dogs with DCM, both mean action potential duration and transmural dispersion of repolarisation increased with LV-epicardial and biventricular pacing. This group proposed that such changes could result in the formation of unidirectional block and re-entry, a phenomenon which is linked to the development of malignant ventricular arrhythmias.

**Figure 3:**
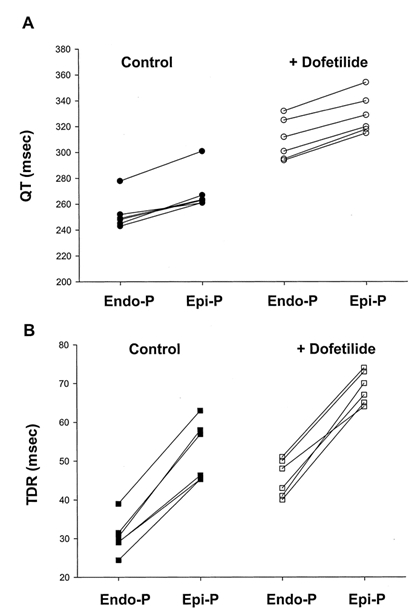
Effects of endocardial pacing (Endo-P) and epicardial pacing (Epi-P) on: (A) QT interval duration and (B) TDR in arterially perfused rabbit left ventricular preparations with and without the administration of dofetilide. Note that epicardial Epi-P led to a more pronounced prolongation in TDR than the QT interval duration. Reproduced with permission from Lippincott Williams & Wilkins [7].

In experiments using isolated arterially perfused rabbit LV wedge preparations, Medina-Ravell et al [7] have recently shown that switching from endocardial to epicardial pacing produces an increase in QT interval and transmural dispersion of repolarisation, without associated increases in ventricular transmembrane action potential durations. Administration of dofetilide, an agent which increases the action potential duration, led to a prolongation of the QT interval and TDR, an effect which was more pronounced with epicardial pacing. (Figure 3) In a further experiment, epicardial pacing was shown to facilitate transmural propagation of early repolarisations, the emergence of R-on-T extrasystoles and torsade-de-pointes ventricular tachycardia.

**Figure 4:**
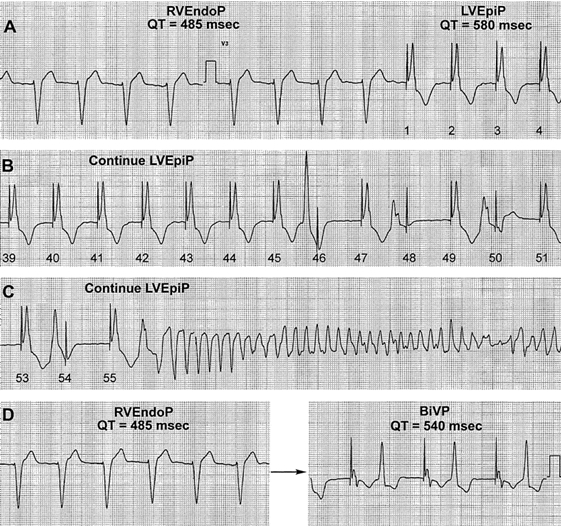
Changes in QT interval, R-on-T ventricular extrasystoles and the development of torsade-de-pointes ventricular tachycardia in response to pacing at different sites. After switching from right ventricular endocardial pacing (RVEndoP) to left ventricular epicardial pacing (LVEpiP), the QT interval increased. Continued LVEpiP led to ventricular extrasystoles (B) and, eventually, to torsade-de-pointes ventricular tachycardia (C). Switching from RVEndoP to biventricular pacing (BiVP) led to an increase in the QT interval duration and to the emergence of R-on-T ventricular extrasystoles. Reproduced with permission from Lippincott Williams  & Wilkins [7].

**Figure 5:**
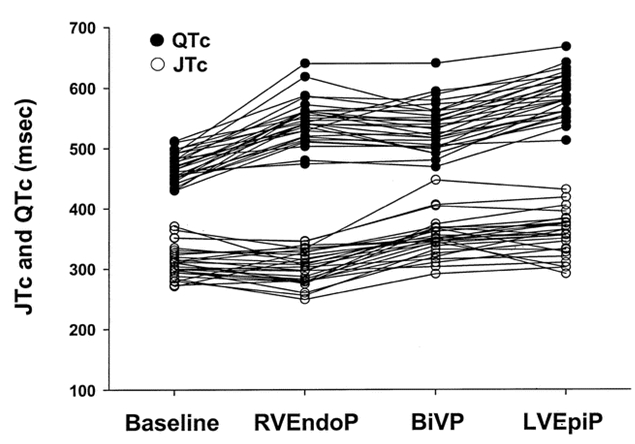
QT interval (●) and JT interval (○) duration during baseline rhythm, right ventricular endocardial, biventricular and left ventricular epicardial pacing in 29 patients with heart failure. Reproduced with permission from Lippincott Williams   & Wilkins [7].

In humans, reversal of the normal sequence of activation has similarly been linked to arrhythmogenesis. Medina-Ravell et al [7] showed that, in one patient, switching from endocardial to epicardial left ventricular pacing, the QT interval increased from 485 to 580 ms, an effect which was associated with the development of torsade-de-pointes ventricular tachycardia. (Figure 4) Switching from right ventricular endocardial pacing to BiVP led to an increase in the T interval followed by R-on-T ventricular extrasystoles. Importantly, a substudy of 29 patients with heart failure showed that LV epicardial pacing and BiVP led to increases in QT, JT and transmural dispersion of depolarisation [7]. (Figure 5) These data suggest that a BiVP- or LV epicardial-dependent increase in the QT interval and transmural dispersion of repolarisation has the potential for increasing the risk for the development of ventricular arrhythmias.

## Cardiac resynchronisation therapy, arrhythmias and sudden cardiac death

Early studies suggested that CRT exerts an antiarrhythmic effect. Paul's group showed that CRT significantly reduced ventricular ectopic counts when compared to isolated right ventricular pacing [24]. In the Ventak CHF trial (single-blinded, randomised, cross over study of CRT-D for 3 months, followed by 3 months of no pacing plus implantable cardioverter defibrillator), Higgins et al observed a lower frequency of antitachycardia therapies during BiVP compared to no pacing (16% v 34% respectively; p=0.04) [25].

Although the CARE-HF Extension study reported a reduction in sudden cardiac death following CRT [26], such an effect was not apparent after the initial 29 months' follow-up (CRT: 35% versus medical therapy alone: 32%) [1]. A meta-analysis of randomised trials of CRT, which did not include the CARE-HF study, showed that while CRT reduces death from progressive heart failure, death from causes other than pump failure may have been increased [27]. A subsequent meta-analysis of randomised trials which did include CARE-HF found no effect from CRT on sudden cardiac death [6]. In balance, it would appear that CRT exerts its beneficial effects on mortality by reducing mortality from pump failure, rather than from arrhythmic events.

Increasing interest is being focused on factors which might predict benefit from CRT. A panoply of echocardiographic studies have explored numerous measures of dyssynchrony [28], but only 3 small studies have included mortality and cardiovascular events as endpoints. None of these echocardiographic measures have been validated against mortality or morbidity and no echocardiographic parameters have been shown to predict arrhythmic events. With respect to the possible value of the ECG prior to implantation, QRS duration is not a predictor of benefit.

**Figure 6:**
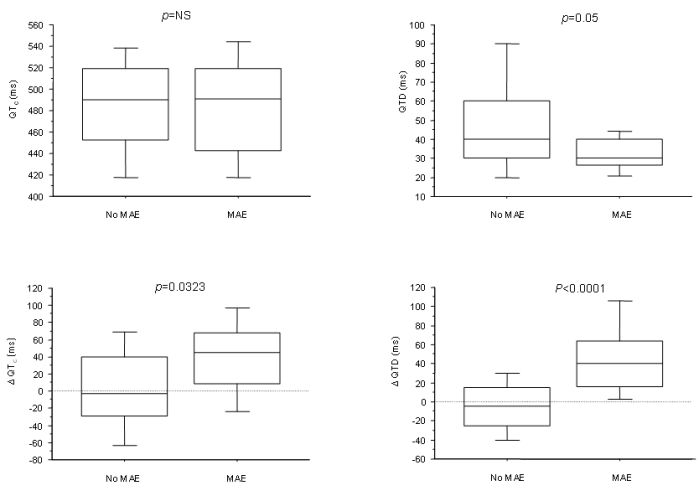
Box plots of baseline QTc interval duration and QT dispersion and their changes from baseline to 48 days following cardiac resynchronization therapy. The five horizontal lines represent the 10^th^, 25^th^, 50^th^, 75^th^ and 90^th^ percentiles of each variable, from bottom to top. Reproduced with permission from Elsevier. [29].

**Figure 7:**
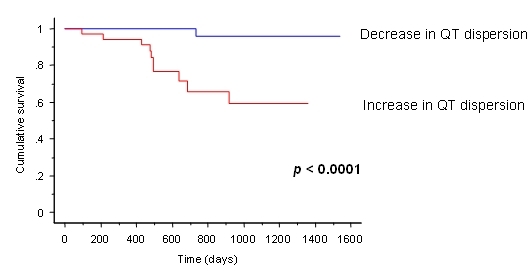
Kaplan-Meier survival curves according to change in QT dispersion (QTD) from baseline to 48 days following biventricular pacemaker implantation. Reproduced with permission from Elsevier [29].

We sought to determine whether QT interval duration and QT dispersion prior to and following implantation predict major arrhythmic events (MAE) following CRT [29]. In a study of 75 patients, 11 patients suffered a MAE over a follow-up of 807 days. Disappointingly, neither the QT interval or QT dispersion prior to implantation predicted MAE. Following CRT, however, a differential effect of CRT on QT dispersion was observed, with 47% of patients exhibiting an increase in QT dispersion above baseline, and 53% showing a decrease. (Figure 6). Major arrhythmic events occurred in 29% of patients exhibiting an increase in QT dispersion and in 3% of those exhibiting a decrease (p=0.0017). In multiple regression analyses, change in QT dispersion from baseline strongly predicted MAE, independently of changes in QTc, QRS duration, left ventricular ejection fraction and end-diastolic volume (p<0.001). Differences in survival curves were observed when patients were dichotomised according to whether QT dispersion increased or decreased in relation to baseline values (p<0.0001). (Figure 7) These findings raise the possibility that CRT has differential effects on the arrhythmogenic substrate, antiarrhythmic in some and arrhythmogenic in others. Similar differential effects on the arrhythomogenic substrate have been observed with other treatments, such as class I antiarrhythmic agents [30].

With regard to the T_peak_-T_end_ interval following CRT, we found that CRT led to an overall reduction in the T_peak_-T_end_ interval, averaging -16.5 ms for a cohort of  75 patients [29]. The reduction, however, was more marked in the no-MAE than in the MAE group (-20.0±5.4 and -1.5±12.8 ms, respectively, p=0.047). Berger et al (31) also found that CRT leads to an overall reduction in T_peak_-T_end_ of 81 ±  13.8 ms (mean ± SD). It would appear from this standard deviation that a reduction in T_peak_-T_end_ interval was not found in all patients. The salient finding from our study is that CRT has a differential effect on the the T_peak_-T_end_ interval and that this is related to the development of MAE.

Our demonstration that CRT is apparently proarrhythmic is some patients is relevant to the observation from the CARE-HF study that CRT reduces all-cause mortality but not sudden cardiac death [1]. Arguably, the favourable effects of CRT on mortality are partly negated by an increase in the risk of death from fatal arrhythmias. Our findings are also relevant to the secondary end-point data from the Comparison of Medical Therapy, Pacing and Defibrillation in Heart Failure  (COMPANION) trial [4], which reported that all-cause mortality was lower in the CRT and CRT-D groups than in the pharmacological group after 12 months (15% and 12% compared to 19%, p=0.059 and p=0.003, respectively). Interestingly, the survival curves for all-cause mortality begin to separate between day 270 and 360 after randomisation. Our finding that pacing-induced increases in QT dispersion becomes apparent around that time, between day 350 and 450 post-implantation, may not be coincidental.

## Modulation of QT dispersion

As discussed above, CRT appears to have a differential effect on QT dispersion, which is related to the risk of developing ventricular tachyarrhythmias. No studies have explored which factors are responsible for this differential effect. Intuitively, patients with worse left ventricular function may be at an increased risk. In this respect, Pai et al have shown that in patients with heart failure, QT dispersion is negatively related to ejection fraction [22]. In a study of 103 patients with ischaemic cardiomyopathy, Bountinoukos et al studied QT dispersion in relation to myocardial viability, assessed using dobutamine stress echocardiography [32].  This group found that QT dispersion was lower in patients with at least 4 viable myocardial segments than in patients with no viability. These findings are supported by those Schinkel et al, who employed single photon emission cardiac tomography for assessing viability [33].

Perfusion may also influence the arrhythmogenic substrate. Bonnemeier et al have shown that QT dynamicity, a temporal measure of QT dispersion, is increased following percutaneous coronary intervention in patients with TIMI 2 flow, compared to patients with TIMI 3 flow [34], suggesting that the arrhythmogenic substrate is potentiated by incomplete revascularisation. The finding that surgical revascularisation reduces QT dispersion [35] is consistent with this finding.

It is well established that in patients with ischaemic [36-38] and non-ischaemic cardiomyopathy [39], re-entrant circuits in the border zone surrounding myocardial scars are the source of ventricular tachycardia. Currently, left ventricular leads during implantation for CRT are placed without regard to the site of myocardial scars. Epicardial pacing in the border zone of a scar may, conceivably, be arrhythmogenic.

Ventricular dyssynchrony may also influence the arrhythmogenic substrate. In the study of Spragg et al [40], adult dogs underwent left bundle branch radiofrequency ablation and tagged MR imaging to confirm left ventricular dyssynchrony. Four weeks later, hearts were excised and myocardial segments were isolated. Interestingly, conduction velocity, action potential duration and refractory period were significantly reduced in the late-activated, lateral wall of dyssynchronous hearts compared to the anterior wall. Moreover, the normal difference in conduction velocity between the endocardial and pericardial layers were reversed in the dyssynchronous lateral wall. The subcellular location of conexin43 was redistributed in late-activated myocardium from intercalated discs to lateral myocyte membranes. The salient finding from this study is that dyssynchrony alone induces regionally specific changes in conduction and repolarisation. Whether or not dyssynchorny is in itself proarrhythmic has not been explored. Nor have any studies focused on the possibility that incomplete resynchronisation might be responsible for increased arrhythmogenesis.

## Conclusions and implications for further research

The incidence of sudden, presumed arrhythmic, deaths in patients with heart failure treated with CRT remains high, comparable to patients receiving pharmacological therapy alone. Both animal and human experimental evidence indicates that reversal of the normal direction of myocardial activation, as it occurs during conventional CRT, promotes arrhythmogenesis. Such findings are consistent with human experiments showing that epicardial pacing leads to increased transmural dispersion of repolarisation and ventricular arrhythmias. Studies of patients undergoing CRT have shown that CRT has a differential effect on QT dispersion, with some patients exhibiting an increase above pre-implant values. Such an increase in QT dispersion carries a risk of arrhythmic events.

Little is known about which factors might modulate the arhythmogenic substrate in patients undergoing CRT. Identification of factors that render some patients susceptible to the putative arrhythmogenic effects of CRT may be useful in patient selection. Further studies are needed to determine whether underlying left ventricular function, dyssynchrony and the site of the LV pacing lead in relation to myocardial scars are relevant in this respect. An alternative solution to the apparent arrhythmogenic effects of CRT is to use LV endocardial pacing, as this would preserve the natural direction of myocardial activation.
